# Early childhood caries in Romania: non-linear and spatial analysis of geographic sugar intake using LOESS, GAMs, and choropleth mapping

**DOI:** 10.3389/froh.2026.1822133

**Published:** 2026-05-12

**Authors:** Ruxandra Sava-Rosianu, Guglielmo Giuseppe Campus, Vlad Tiberiu Alexa, Antonia Ilin, Mariana Postolache, Daniela Jumanca, Roxana Oancea, Ruxandra Sfeatcu, Mariana Caramida, Alexandrina Muntean, Daniela Esian, Alice Murariu, Constantin Daguci, Octavia Balean, Vanessa Bolchis, Berivan Laura Rebeca Buzatu, Delia Luca, Nicoleta Toderas, Atena Galuscan

**Affiliations:** 1 Translational and Experimental Research Center in Oral Health, “Victor Babes” University of Medicine and Pharmacy Timisoara, Timisoara, Romania; 2 Department of Cariology, Institute of Odontology at Sahlgrenska Academin, University of Gothenburg, Gothenburg, Sweden; 3 Department of Dental Sciences and Maxillo-Facial Surgery, Sapienza University of Rome, Rome, Italy; 4 Department of Cariology, Saveetha Dental College and Hospitals, SIMATS, Chennai, India; 5 Department of Public Health, University of Medicine and Pharmacy Bucharest, Bucharest, Romania; 6 Department of Oral Health and Community Dentistry, Faculty of Dentistry, “Carol Davila” University of Medicine and Pharmacy, Bucharest, Romania; 7 Department of Pedodontics, Faculty of Dental Medicine, University of Medicine and Pharmacy “Iuliu Hatieganu”, Cluj-Napoca, Romania; 8 Department of Pedodontics, Faculty of Dental Medicine, University of Medicine and Pharmacy, Targu Mures, Romania; 9 Department of Community Dentistry, Faculty of Dental Medicine, University of Medicine and Pharmacy “Grigore T. Popa”, Iasi, Romania; 10 Department of Oro-Dental Prevention, Faculty of Dental Medicine, University of Medicine and Pharmacy of Craiova, Craiova, Romania; 11 Specialization in Clinical Psychology and Psychotherapy, Department of Psychology, Faculty of Sociology and Psychology, West University of Timisoara, Timisoara, Romania

**Keywords:** early childhood caries, generalized additive models, non-linear mapping, spatial mapping, sugar consumption

## Abstract

**Background:**

Excessive intake of free sugars in early childhood is a major modifiable risk factor for early childhood caries (ECC). Evidence on geographic variation in sugar consumption among preschool children remains limited, particularly in Eastern Europe.

**Objectives:**

To assess patterns of sugar intake among Romanian preschool children and to explore non-linear and spatial associations between sugar consumption and geographic areas using advanced statistical and mapping techniques.

**Methods:**

Baseline data from the Smilebright Romania oral health programme were analyzed for 391 children aged 4–5 years. Parental questionnaires assessed the frequency of consumption of natural and processed sugars using a Likert scale. Sugar intake indices (total, processed, and natural) were derived as mean frequency scores. Non-linear associations between sugar intake and geographic variables were explored using locally weighted scatterplot smoothing (LOESS) and tested using generalized additive models (GAMs). County-level mean intake values were visualized using choropleth maps.

**Results:**

At the macro-regional level, significant variation was observed only for natural sugar intake (edf = 1.69, F = 7.32, *p* < 0.01), while processed and total sugar intake showed no significant differences. At the county level, significant geographic variation was identified for total, processed, and natural sugar intake (all *p* < 0.01), although adjusted R^2^ values were modest (0.04–0.07). Spatial mapping revealed higher levels of total and processed sugar intake predominantly in north-eastern counties, whereas natural sugar intake was higher in south-western regions.

**Conclusions:**

Sugar intake among Romanian preschool children demonstrates non-linear and spatially structured geographic patterns, particularly at finer geographic resolution. Although geographic factors explain a limited proportion of variability, their consistent influence supports the value of spatially informed analyses. Integrating flexible statistical modeling with geographic visualization can enhance understanding of dietary risk factors relevant to ECC prevention and inform targeted public health strategies details.

## 1 Introduction

Oral diseases were explicitly and extensively referenced in the Political Declaration of the Fourth UN High-Level Meeting (HLM4) on NCDs. This was a historic milestone resulting from sustained advocacy by organizations like the FDI World Dental Federation and the International Association for Dental, Oral and Craniofacial Research (IADR) (1).

Oral diseases and conditions share many of the same risk factors as major noncommunicable diseases (NCDs), including tobacco use in all its forms, harmful alcohol consumption, excessive intake of free sugars, and insufficient exclusive breastfeeding (2–5).

The global prevalence of oral diseases and conditions constitutes a pressing public health concern, given the substantial social, economic and environmental consequences it entails. These diseases disproportionately impact poor, vulnerable, and marginalized populations, including individuals with low incomes, those living with disabilities, older adults residing alone or in care facilities, as well as refugees, incarcerated individuals, people in remote or rural areas, and members of minority or other socially disadvantaged groups (5, 6).

In Romania, the first national oral health survey showed a very high caries prevalence in children with a mean dmft index of 4.89 and a significant caries index (SIC) of 9.83 for six-year-olds, and a DMFT of 4.96 for 12-year-olds (7). These findings must be interpreted in the context of limited access to dental care services, as the majority of oral health services are delivered through the private sector, creating financial and geographic barriers to care. In addition, there is no comprehensive national prevention strategy, and community-based fluoridation programs are not implemented, further reducing opportunities for population-level caries control. Within this framework, the mean dmft index was found to be strongly correlated with socioeconomic status and parental educational level, highlighting the persistent inequalities in oral health and the need for targeted public health interventions.

The World Health Organization (WHO) recommends that free sugars account for less than 10% of total energy intake in both children and adults (8).

The American Heart Association and the International Association of Paediatric Dentistry (IAPD) recommend limiting sugar intake during early childhood, advising that no free sugars be provided to children under two years of age (9–13).

Free sugars are defined as all monosaccharides and disaccharides added to foods by manufacturers, cooks, or consumers, as well as sugars naturally present in honey, syrups, fruit juices, and fruit juice concentrates (8).

Diet is a major modifiable factor contributing to the development of dental caries. Frequent and prolonged exposure of teeth to fermentable carbohydrates, particularly sugars, creates an acidic plaque environment that promotes enamel demineralization and caries formation (14). Consequently, diets high in sugar are associated with an increased risk of caries, whereas sugar restriction is recognized as a key preventive strategy.

Two primary dietary approaches have been proposed to reduce sugar intake for caries prevention: decreasing the total amount of added sugar consumed or reducing the frequency of sugar intake. Distinguishing the relative importance of these two factors is challenging, as an increase in one often coincides with an increase in the other. Several studies have suggested that the frequency of sugar consumption, rather than the total amount, is more strongly associated with caries development (15, 16). For example, Palmer et al. classified foods based on cariogenic potential and reported that frequent intake of highly cariogenic foods or between-meal juices was linked to early childhood caries (ECC) in US children aged 2–6 years (17). Similarly, Hong et al. found that frequent consumption of foods and drinks containing added sugar was associated with caries among UK children aged 12–15 years (18). Zeng et al. reported that the frequency of sugary snack and drink consumption, as well as snacking before sleep, was correlated with caries development in Chinese children aged 3–5 years (19).

Other risk factors for dental caries and severe periodontal diseases include inadequate oral hygiene. These conditions are also influenced by commercial determinants—strategies employed by certain private-sector actors to promote products and behaviors that are harmful to health. Such strategies encompass the marketing, advertising, and sale of products linked to oral diseases, including tobacco and foods and beverages high in free sugars (1).

Most oral diseases are largely preventable and can be effectively addressed through population-level public health measures. Downstream interventions remain essential, including preventive measures and the provision of evidence-based clinical oral health care. Upstream policy interventions targeting social and commercial determinants are particularly cost-effective and have broad population reach and impact. Midstream initiatives focus on creating supportive environments in key settings, such as households, schools, workplaces, long-term care facilities, and community venues (2, 6, 20, 21)

The Romanian school system is structured in successive levels regulated by national education law. It begins with early childhood education (ante-preschool and preschool), followed by primary education (grades 0–4), lower secondary education or gymnasium (grades 5–8), and upper secondary education, which includes high school and vocational education (grades 9–12 or 13). Preschool education is part of early childhood education and typically serves children aged 3 to 6 years. It is organized in kindergartens, structured into three age-based groups: the small group (ages 3–4), middle group (ages 4–5), and large group (ages 5–6). Attendance is voluntary for younger children, but the last two years are compulsory, as it prepares children for entry into primary school.

The present paper was designed to describe the ECC data collected from the baseline observation of the Smilebright project. This program was designed with a focus on the early diagnosis of carious lesions through comprehensive clinical examinations conducted within nurseries. It aimed to address risk factors by assessing oral health-related knowledge and behaviors, coupled with health education initiatives. Additionally, it evaluated the efficacy of halting the progression of caries through the application of fluoride toothpaste. Furthermore, it has established new primary care teams—comprising dental hygienists—thereby providing a robust framework for the integration of dental caries prevention and control within broader health interventions. The paper has the aim to assess prevalence data on ECC with background factors with a main focus on dietary habits using non-linear and spatial mapping techniques.

## 2 Material and methods

For This study reports data on caries prevalence and dietary habits among Romanian children aged 4–5 years that were enrolled to take part of the Smilebright Romania child oral health programme. Ethical approval was obtained from the Department of Scientific Research Ethics, University of Medicine and Pharmacy “Victor Babeș,” Timișoara, Romania (approval number 59/17.12.2023, revised 2024). Participation was voluntary, and informed consent was obtained from all participants, who retained the right to withdraw at any time. The programme received approval from the Romanian Ministry of Health (AFR2089/DGAM 1014). The study protocol is registered at ClinicalTrials.gov under Protocol NCT06441500 (registered 28 May 2024) and was published in 2025 (22).

### 2.1 Sample size calculation and sample selection

Sample size was calculated in accordance with WHO guidelines, using a stratified sampling approach.

Romania has a population of approximately 19 million inhabitants, characterized by a balanced but heterogeneous distribution between urban and rural areas. Roughly 55%–60% of the population resides in urban centers, while a substantial proportion (40%–45%) lives in rural communities, many of which are geographically dispersed and have limited access to healthcare services. The country encompasses diverse geographic regions (including mountainous, hilly, and plain areas) that contribute to variations in infrastructure, socioeconomic development, and healthcare accessibility. This demographic and territorial heterogeneity is associated with marked disparities in income levels, educational attainment, and access to medical and dental services, particularly between urban and rural populations. Consequently, ensuring representativeness in epidemiological studies requires proportional inclusion of participants from both settings, as well as from different geographic regions. Such an approach allows for a more accurate reflection of the national population structure and strengthens the external validity of findings related to oral health outcomes and associated risk factors. The country is divided into 5 macroregions (central, south-west, south-east, north-west and north-east) 40 counties (administrative units), with Bucharest considered separately as the capital. Administrative units are further classified into micro-regions, metropolitan areas (population >250,000), cities (100,000–250,000), small cities (<100,000), and villages (<5,000).

To achieve a nationally representative sample, publicly available data from 4,696 schools across all 41 administrative units (40 counties plus Bucharest) were analyzed via County School Inspectorate websites to understand the territorial distribution of the target population. For each county, the total number of pupils and the proportion of children in each administrative unit were calculated. These proportions were used to estimate the number of children to be included per county, further stratified by locality type (urban vs. rural).

The final sample size was 460 children aged 4–5 years, calculated using the following parameters: type I error of 0.05 (z₁-*α*/2 = 1.96), power of 0.9 (z₁-*β* = 1.28), standard deviation (SD) = 2, design effect = 2, and a 10% anticipated drop-out rate. Sample size calculations for odds ratios were based on expected mean percentage differences between groups, as recommended in the literature for interventions evaluating caries prevalence or increment, including fluoride varnish, oral hygiene instruction, motivational techniques, and oral health education (16–18). Demographic stratification considered regional distribution and age. The studied areas were divided in accordance with the level 1 of NUTS 2024 European statistical atlas. Urban and rural nurseries were randomly selected within each county using the randomization function in MS Excel.

Inclusion criteria were enrollment in kindergarten, parental consent, and child assent. Exclusion criteria included chronic systemic diseases, history of allergic reactions, the presence of fluorosis or other enamel defects, and uncooperative behavior during assessment.

### 2.2 Data collection

#### 2.2.1 Self assessment

Pre-intervention baseline data were collected following the WHO Stepwise Approach, using a self-assessment method. Information on children's oral health behaviors—including dietary habits, frequency of toothbrushing, use of fluoridated toothpaste, use of additional oral hygiene aids (e.g., interdental brushes, mouthwash), frequency of dental check-ups, and reason for the last dental visit as well as potential confounders like socioeconomic status, parental educational level, and urban–rural residence—was obtained using the previously developed National Oral Health Survey questionnaire (study protocol). Responses were provided by parents or caregivers prior to the clinical examination. In cases where family members or caregivers were unable to read the questionnaire, assistance was provided by kindergarten educators.

#### 2.2.2 Clinical examination

Clinical examinations were conducted by calibrated medical staff from each partner university using the International Caries Detection and Assessment System (ICDAS) criteria (23). Examiner calibration followed the International Caries Classification and Management System (ICCMS) guidelines, with intra- and inter-examiner reliability assessed using kappa statistics. The observed level of agreement was above 88%.

Examinations were performed in the kindergarten setting, with children seated, using a dental mirror and probe, without air-drying. Consequently, ICDAS codes 1 and 2 were merged and recorded as category “A.”

### 2.3 Statistical analysis

Data analysis was performed with R studio Version 2024.12.1 + 563, using the mgcv, ggplot2, sf, naturalearth, geodata, haven, tidyverse, kableExtra, janitor, stringi, stringdist and scales packages.

Generalized additive models (GAMs) were specified using penalized regression splines to allow flexible, non-linear relationships between sugar intake (continuous outcome) and geographic predictors (e.g., latitude, longitude, and regional indicators). Models were fitted using the mgcv package in RStudio, with smoothing parameters estimated via restricted maximum likelihood (REML). The general form of the model was:

$$Y_{i}=\beta_{0}\;+\;s(X_{i})+\varepsilon_{i}$$

where Y_i_ represents the sugar intake index, s(X_i_) is a smooth function of the geographic predictor, and ɛ_i_ is the error term assumed to be normally distributed.

To account for the hierarchical structure of the data (children nested within counties), clustering was addressed by including county as a random effect in the GAM framework (i.e., mixed-effects GAM), thereby adjusting for intra-county correlation and potential contextual influences.

Missing data were handled using a complete-case analysis approach, under the assumption that data were missing at random (MAR). The proportion of missingness was low (<5% for all variables), and no systematic patterns were identified. Sensitivity analyses comparing complete-case results with imputed datasets (using multiple imputation via chained equations) showed no meaningful differences, supporting the robustness of the findings.

Sugar intake was assessed trough a Likert scale for each type of sugar ingested, with regards to frequency of consumed fresh fruits, jam, honey, biscuits, cakes, cremes, pancakes, sodas, sweetened chewing gum, candies, sweets, milk/tea/cocoa with sugar or honey. Frequency was graded on 5 levels, as “never”, “several times per month”, “several times per week”, “daily”, “several times per day”. The variable sugar intake was understood as the global mean of the frequency for all of the types of sugar ingested, whereas natural sugar intake refers to sugar that is naturally found in sweet foods, like fresh fruit, milk or natural dairy products and processed sugar intake refers to all of the types of artificially sweetened foods (sweets, candies, bakery products, sweetened beverages). For the microregion loess analysis, NUTS 2024 level 3 was used as a guideline.

Because the relationship between sugar intake and geographical variables was not expected to be strictly linear, locally weighted scatterpoint smoothing LOESS was used to explore the form of association between each outcome (sugar intake) and each predictor (geographic variables). For each outcome variable (total sugar intake, processed sugar intake and natural sugar intake), separate LOESS models were fitted as a function of the predictor variables (main area and county). The smoothing parameter span was set to 0.75, allowing for moderate smoothing of local trends. If the predictor variable would have exhibited a limited number of unique values (less than four) the LOESS procedure would have been adapted by introducing a small random perturbation (jitter) to the predictor and reducing the polynomial degree from quadratic to linear. These adaptive mechanisms were not triggered. All predictors used in the models had a sufficient number of unique values (four main areas and eleven counties), as a result all fitted models retained the default quadratic specification (degree of 2).

## 3 Results

**Figure 1.**
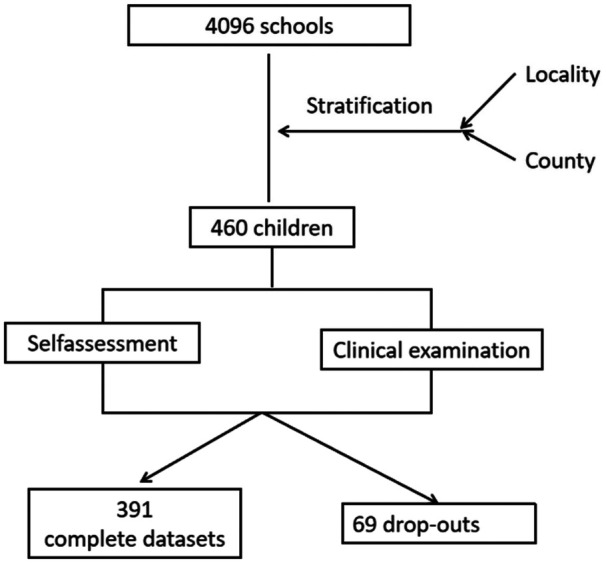
Flow diagramm of participants.

A total of 391 participants were incorporated into the present analysis, owing to the absence of information from the remaining examined children (Figure 1).

Among them, 50.4% (*n* = 197) were male and 49.6% (*n* = 194) were female, with a predominant majority being four years old (50.6%, *n* = 198) at the time of the data collection process. Tooth brushing frequency was assed trough a Linkert scale with the majority of respondent brushing their teeth once a day (41.8%, *n* = 163) followed by twice or multiple times a day 26.9% (*n* = 105), a couple of times a week 13.6% (*n* = 53) once a week with 3.3% (*n* = 13) as seen in Table 1.

**Table 1.** Population description regarding, sex, age and toothbrushing habits.

Variable		%	Mean	Std. dev.	Variance	Variable	Mean	St. dev.	Variance
	
Sex	Female	49.6				Tooth brushing frequency	4.95	0.98	0.97
	Male	50.4							
Age	3	15.6	4.21	0.75	0.56				
	4	50.6							
	5	28.1							
	6	4.1							

**Figure 2.**
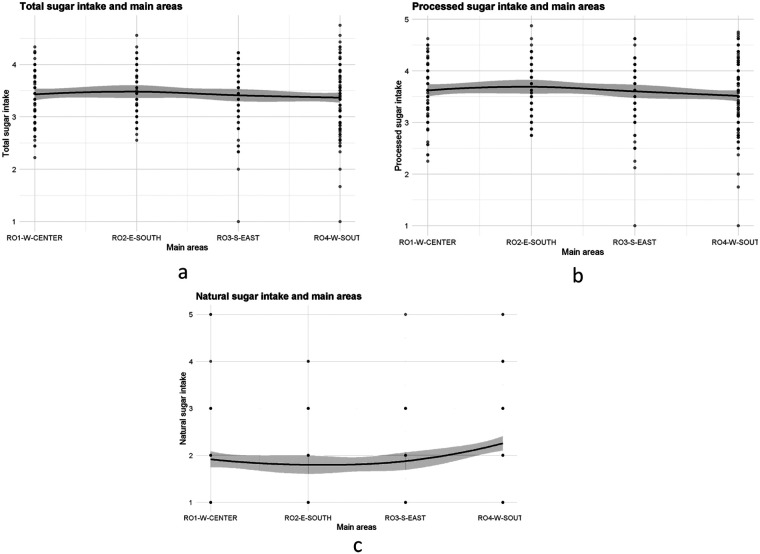
Self reported sugar consumption of the studied population: a) total amount of sugar across the main geographical areas; b) processed sugar consumption across the main geographical areas; c) natural sugar consumption across the main geographical areas.

The LOESS curves for the different types of sugar intake when taken as a function of the four main areas show different patterns. In regards to the natural sugar intake, the curve indicates clear differences between macro-regions, with higher mean values in west-south main region of Romania and lower values in west-center of Romania, the confidence band around the curve remains approximately narrow, suggesting stability in the observed pattern. For processed sugar intake and total sugar intake, the LOESS curves are comparatively flat, with small deviations between regions suggesting more homogeneous levels of processed and total sugar intake across macro regions (Figure 2).

The GAMs tests in relation to the four main areas for natural sugar intake were statistically significant, with the effective degrees of freedom edf = 1.69, with the F value test being F = 7.32, *p* < 0.01. The adjusted R^2^ = 0.03 suggests that macro regions explain in a small but non-negligible proportion the variability in the natural sugar intake. Whereas for processed sugar intake, the effect of main areas didn't reach the levels of statistical significance, same goes for the total sugar intake regarding the main areas (Table 2).

**Table 2:** GAMs for main areas.

Predictor	edf	F	p	R^2^ adj.
Total sugar intake	0.12	0.06	0.28	0.004
Processed sugar intake	0.72	0.88	0.10	0.004
Natural sugar intake	1.69	7.32	0.003	0.038

**Figure 3.**
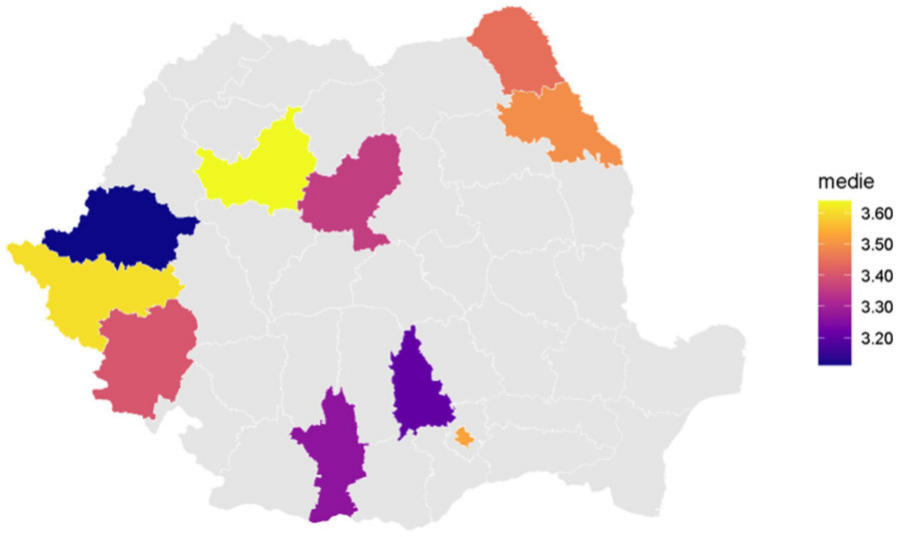
Total sugar intake by county.

In regards to the geographical areas assessed as level 3 NUTS (counties) to smooth the relationship between intake and county, counties were ordered by their mean for the variable of interest. For example, when ordering by the mean for total sugar intake counties with lower mean intake were Arad, Dambovita, Olt whereas counties with higher mean intake were Bucuresti, Timis, Cluj. The LOESS curves of the outcomes for each type of sugar intake shows a monotonic function increase in mean level from low to high intake coutnies. As well as a slight curvature in some cases, indicating that the increase is not perfectly linear, which is to be expected from the edf scores greater than 1 in the corresponding GAMs (Figure 3).

The GAMs tests in relation to the counties for total sugar intake the test is highly significant with an edf=1.42, F = 2.79, *p* < .001, with adjusted R^2^ of about 0.05, indicating that the county level differences explain a small to moderate fraction of the variation in total sugar intake.

For processed sugar intake, the test is statistically significant with an edf = 1.49, F = 3.36, *p* < 0.001, with the adjusted R^2^ equaling to 0.06, which means that the geographical area explains in a small to moderate fraction the variability in the processed sugar intake. For natural sugar intake, a similar pattern emerges, where the test is significant with an edf=1.42, F = 2.34, *p* < 0.001, with adjusted R^2^ around 0.04, explaining in a small amount the variation in natural sugar intake (Table 3).

**Table 3:** GAMs for counties.

Predictor	edf	F	p	R^2^ adj.
Total sugar intake	1.42	2.79	0.001	0.057
Processed sugar intake	1.49	3.36	0.001	0.068
Natural sugar intake	1.421	2.346	0.001	0.048

Choropleth maps depicting county-level means of total, processed and natural sugar intake reveal the spatial patterns of consumption in Romania in children as small as 3-year-old. Counties with higher mean of the total sugar intake appear predominantly in north-eastern part of Romania, whereas counties with lower means of total sugar intake appear to be predominantly in the southern region and south-west region of the country.

In regards to the processed sugar intake, the higher consumption is also in the north east part of the country, with the weaker mean of intake in the south-west part of the country.

**Figure 4.**
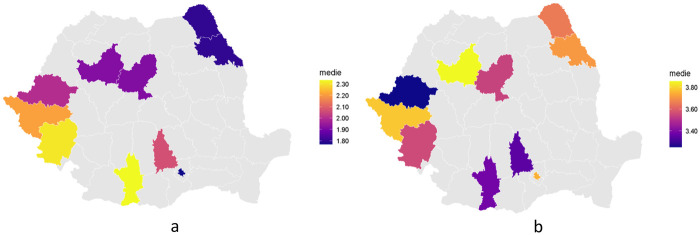
Natural (a) and processed (b) sugar intake by county.

For natural sugar intake, the map highlights a relative reverse compared to the other two maps, with elevated levels of intake in the south west part of the country and lower levels in the north east counties. The counties without available data or not enough of it appear in a neutral grey color (Figure 4).

In regards to the macro regions, natural sugar intake shows statistically significant variation, whereas processed and total sugar intake do not. Even for natural sugar intake, the adjusted R^2^ values are modest, suggesting that the differences exist but account for only a limited share of overall variability in intake. At the level of counties, there are statistically significant differences in all three types of sugar intake. However, the adjusted R^2^ values remain relatively low, indicating that county membership, while significant, accounts for only a small part of the total variability in sugar consumption at a young age.

## 4 Discussions

This study provides novel insight into dietary sugar intake patterns among Romanian preschool children by integrating non-linear statistical modeling with spatial analysis.

Given the absence of strong theoretical or empirical evidence supporting a strictly linear association between sugar intake and geographic variables, this study adopted a flexible analytical strategy. Locally weighted scatterplot smoothing (LOESS) was first employed to explore the functional form of the relationships between sugar intake indices and geographic predictors. The methods used, combined use of LOESS smoothing, generalized additive models (GAMs), and choropleth mapping, offers a comprehensive framework for exploring complex dietary behaviors relevant to early childhood caries (ECC). This exploratory step allowed the identification of potential non-linear patterns, including threshold effects and regional gradients, without imposing restrictive parametric assumptions. Such flexibility is particularly important in nutritional epidemiology, where dietary behaviors are shaped by complex social, cultural, and environmental factors that may vary geographically.

At the macro-regional level, statistically significant variation was observed only for natural sugar intake, whereas processed and total sugar intake appeared relatively homogeneous across regions. This may imply that large-scale regional differences in Romania may reflect cultural or agricultural practices influencing fruit consumption rather than disparities in access to or marketing of processed sugary products. In contrast, county-level analyses revealed significant differences for all sugar intake indices, indicating that finer geographic resolution is more informative for identifying dietary inequalities in young children.The findings were in accordance with the results of earlier studies in children in other countries with different cultures or socio-economical levels (17–19, 24, 25).

The findings indicate that, although statistically significant geographic variation in sugar intake is present, the overall predictive power of the main exposure variable—regional or county-level classification—remains limited. Specifically, the low adjusted R^2^ values suggest that macro-regional or county membership explains only a small proportion of the variability in children's sugar consumption. This implies that, while geographic patterns can be detected, they are not strong determinants of intake at the individual level and should be interpreted with caution when informing targeted public health strategies.

This modest explanatory capacity likely reflects the multifactorial nature of dietary behaviors in early childhood. Key unmeasured or residual confounders may play a more substantial role, including household socioeconomic status, parental educational level, nutritional knowledge, and cultural feeding practices. In addition, factors such as food availability, marketing exposure, school food environments, and urban–rural differences may further influence sugar consumption patterns beyond administrative geographic boundaries.

Potential sources of bias should also be considered. The reliance on questionnaire-based data introduces the possibility of recall bias and social desirability bias, particularly in parental reporting of children's dietary intake. Selection bias may have occurred due to the exclusion of participants with incomplete responses or absence at the time of examination, potentially affecting representativeness. Furthermore, measurement limitations—such as the categorization of sugar types or lack of quantitative dietary assessment—may have attenuated observed associations.

To formally evaluate the statistical significance of the associations suggested by LOESS curves, generalized additive models (GAMs) were subsequently applied. GAMs offer a robust framework for modeling non-linear relationships through smooth functions, enabling confirmation of observed trends while retaining interpretability. Although few studies have applied both LOESS and generalized additive models to dietary intake and geographic predictors specifically, analogous work in spatial health research demonstrates the value of these methods. For instance, semi-parametric geo-additive models using LOESS smoothers have been applied to map spatial variation in anemia risk, capturing non-linear geographic trends that traditional models may overlook (26).

Similarly, generalized additive models have been used to smooth geocoded environmental and socioeconomic exposures in relation to disease outcomes, revealing spatial heterogeneity in health risks (27, 28). Despite statistical significance, the adjusted R^2^ values across GAMs were modest, indicating that geographic location explains only a limited proportion of variability in sugar intake. This finding aligns with existing evidence that dietary behaviors in early childhood are multifactorial and strongly influenced by household-level determinants such as parental education, feeding practices, socioeconomic status, and food environment (29). Nonetheless, even small geographic effects may have meaningful public health implications when they consistently affect large populations, particularly in the context of ECC, which is highly sensitive to early dietary exposures.

Compared with dietary studies that map regional patterns without formal smoothing, the combined use of LOESS for exploratory visualization and GAMs for formal inference adds analytical depth, allowing more nuanced detection and testing of geographic gradients in sugar intake.

The effective degrees of freedom (edf) provided insight into the complexity of each association, with values close to one indicating near-linear effects and higher values reflecting increasingly non-linear relationships. The reporting of F statistics and corresponding *p* values for the smooth terms allowed hypothesis testing of these associations, strengthening the inferential validity of the findings beyond purely descriptive analyses. Another study on the comparative effectiveness of school-based caries prevention used generalized additive models to assess non-linear trends in dental caries outcomes over time. In this study, GAMs were used to estimate smoothed effects of age and visit (time) on total observed caries experience (TOCE) and untreated decay in children receiving different caries prevention programs (30). Effective degrees of freedom (edf) were reported for smooth terms, where higher edf indicated departures from linearity (e.g., edf > 1 for non-linear effects on TOCE across visits), and F-statistics with *p* values evaluated the significance of these smooth trends.

Earlier methodological work in dentistry has highlighted the conceptual usefulness of GAMs to model complex relationships between predictors and caries, but did not routinely report formal edf or hypothesis testing (31). Our application advances this tradition by systematically reporting edf to quantify non-linear relationships between sugar intake and geographic variables and using *F* tests to formally evaluate these smooth terms. This strengthens the inferential validity of our findings, aligning with best practices in flexible regression while extending their use to spatial dietary epidemiology.

Model performance was assessed using adjusted R^2^ as an approximate measure of effect size. Although adjusted R^2^ values in GAMs are not directly comparable to those obtained from linear regression models, they nonetheless offer a useful indication of the proportion of variability in sugar intake explained by geographic predictors. In a public health context, even modest effect sizes may be meaningful, particularly when non-linear associations are consistent across multiple sugar intake indices or geographic dimensions. These findings underscore the relevance of geographic context as a contributing factor to dietary patterns at the population level.

To complement regression-based analyses, county-level means for each sugar intake index were calculated and integrated with geographic shapefiles of Romanian counties to produce choropleth maps. This spatial visualization approach facilitated the identification of regional disparities in sugar consumption and provided an intuitive representation of geographic patterns. Choropleth mapping serves as a valuable descriptive tool, enabling the detection of clusters of higher or lower sugar intake that may not be readily apparent from statistical models alone. Use of spatial visualization through geographic maps has a well-established precedent in oral epidemiology. For example, geo-mapping of early childhood caries risk in Sweden revealed clear parish-level disparities in caries burden, supporting targeted prevention programs (32). Similarly, studies from Brazil and India have employed GIS tools to visualize the geographic distribution of DMFT indices and caries prevalence across urban districts or wards, facilitating intuitive identification of high-burden areas and spatial clustering (33, 34). In line with these approaches, the choropleth maps of county-level sugar intake indices in our study provide a visual summary of regional differences in dietary behavior that may not be fully captured by regression models alone. Such thematic maps serve as valuable descriptive tools in spatial epidemiology, enabling policymakers and stakeholders to see geographic patterns and prioritize areas for intervention.

However, the spatial analyses must be interpreted with caution. Aggregation at the county level introduces the potential for ecological fallacy, as observed group-level patterns may not reflect individual-level behaviors. Additionally, the modifiable areal unit problem (MAUP) may influence the observed spatial distributions, as different geographic aggregation schemes could yield alternative patterns. Within-county heterogeneity in dietary behaviors may also be obscured by the use of mean values, particularly in counties characterized by pronounced urban–rural contrasts.

Despite these limitations, the combined use of LOESS, GAMs, and geographic information system–based visualization represents a methodological strength. This integrated approach allows exploratory pattern detection, formal statistical testing, and spatial contextualization within a single analytical framework. The consistency of findings across these complementary methods enhances confidence in the robustness of the observed associations.

From a public health perspective, the identification of non-linear and spatially structured patterns in sugar intake has important implications. Geographic variation suggests that uniform national strategies may have differential effectiveness across regions. Spatially informed analyses can support the development of region-specific interventions, educational campaigns, and policy measures aimed at reducing excessive sugar consumption. Moreover, the methodological framework employed in this study is transferable and could be applied to other dietary risk factors or health outcomes to inform targeted prevention strategies.

## 5 Conclusion

In summary, this study highlights geographically structured, non-linear patterns of sugar intake among Romanian preschool children and underscores the added value of flexible statistical and spatial methods in oral health research. While geographic factors explain only a modest share of variability in sugar consumption, future studies should incorporate a broader set of individual-, household-, and environment-level variables, ideally using multilevel modeling approaches, to better capture the complex determinants of dietary behaviors and improve the explanatory power of analytical models.

## Data Availability

The raw data supporting the conclusions of this article will be made available by the authors, without undue reservation.
